# The Basis and Promise of Programmable RNA Editing and Modification

**DOI:** 10.3389/fgene.2022.834413

**Published:** 2022-01-28

**Authors:** Nicholas Lo, Xin Xu, Fraser Soares, Housheng Hansen He

**Affiliations:** ^1^ Princess Margaret Cancer Center, University Health Network, Toronto, ON, Canada; ^2^ Department of Medical Biophysics, University of Toronto, Toronto, ON, Canada

**Keywords:** RNA editing, base editing, M6A, epitranscriptome, CRISPR

## Abstract

One key advantage of RNA over genomic editing is its temporary effects. Aside from current use of DNA-targeting CRISPR-Cas9, the more recently discovered CRISPR-Cas13 has been explored as a means of editing due to its RNA-targeting capabilities. Specifically, there has been a recent interest in identifying and functionally characterizing biochemical RNA modifications, which has spurred a new field of research known as “epitranscriptomics”. As one of the most frequently occurring transcriptome modifications, N6-methyladenosine (m6A) has generated much interest. The presence of m6A modifications is under the tight control of a series of regulators, and the ability of fusing these proteins or demethylases to catalytically inactive CRISPR proteins have resulted in a new wave of programmable RNA methylation tools. In addition, studies have been conducted to develop different CRISPR/Cas and base editor systems capable of more efficient editing, and some have explored the effects of *in vivo* editing for certain diseases. As well, the application of CRISPR and base editors for screening shows promise in revealing the phenotypic outcomes from m6A modification, many of which are linked to physiological, and pathological effects. Thus, the therapeutic potential of CRISPR/Cas and base editors for not only m6A related, but other RNA and DNA related disease has also garnered insight. In this review, we summarize/discuss the recent findings on RNA editing with CRISPR, base editors and non-CRISPR related tools and offer a perspective regarding future applications for basic and clinical research.

## Introduction

Genome editing and modification technologies such as transcription activator-like effector nucleases (TALENs) and zinc-finger nucleases (ZFNs) originated from earlier nuclease technologies and other chemical techniques. However, these earlier methods were limited in terms of editing specificity and riddle with off-target side effects (Pattanayak et al., 2011). When the introduction of the bacteria-derived RNA-guided clustered regularly interspaced short palindromic sequences (CRISPR-Cas9) system was discovered, this new technique changed the versatility of genome editing (Gasiunas et al., 2020). An explosion of other CRISPR/Cas systems since then have been characterized and provided a molecular toolbox for basic and translational research. More recently, the application of systems such as CRISPR-Cas13—used by bacteria to degrade viral RNA, has opened a new area of exploration for editing techniques and is currently being adapted for uses in mammalian species (Cox et al., 2018).

Base editing is a method currently used to introduce single nucleotide variants into DNA or RNA (Porto et al., 2020). Different components of CRISPR systems and other proteins (e.g., deaminases) come together to make point mutations without introducing double-strand breaks. The direct base changes limit the number of byproducts, making them a potential therapeutic option for future applications (Sun and Yu, 2019). The utility of these and other CRISPR/Cas systems to investigate the epitranscriptome has become an emerging area that aims to identify and functionally characterize biochemical modifications on RNA. Specifically, N6-methyladenosine (m6A) modifications appear abundantly on mRNA and non-coding RNA that are often involved in regulatory processes such as splicing, translation, and RNA stability (Chen et al., 2019). The effects and presence of m6A are mediated by three main classes of proteins: readers, erasers, and writers, which can be bound as an additional domain to existing Cas-related systems allowing for the development of programmable RNA methylation tools. Here, the characteristics of current base editing systems and a few CRISPR/Cas systems are analyzed in order to describe their utility for understanding RNA modifications such as m6A.

### CRISPR/Cas Systems

When CRISPR systems emerged, the advantages over TALENs and ZFNs were observable. CRISPR-Cas9 is capable of editing with higher efficiency and precision on DNA at multiple loci simultaneously. It is able to target a given genome sequence through modifying the guide RNA sequence, whereas TALENs and ZFNs require the re-coding of proteins for each new target site (Gupta and Musunuru, 2014). Importantly, its smaller size allowed for easier cell delivery, as the bulky size of TALENs’ cDNA showed to be a hindrance, limiting its therapeutic applications. One of the more notable advantages was the accessible design, allowing greater use at a lower price, and thus making it more practical for larger-scale applications such as screening.

**FIGURE 1:**
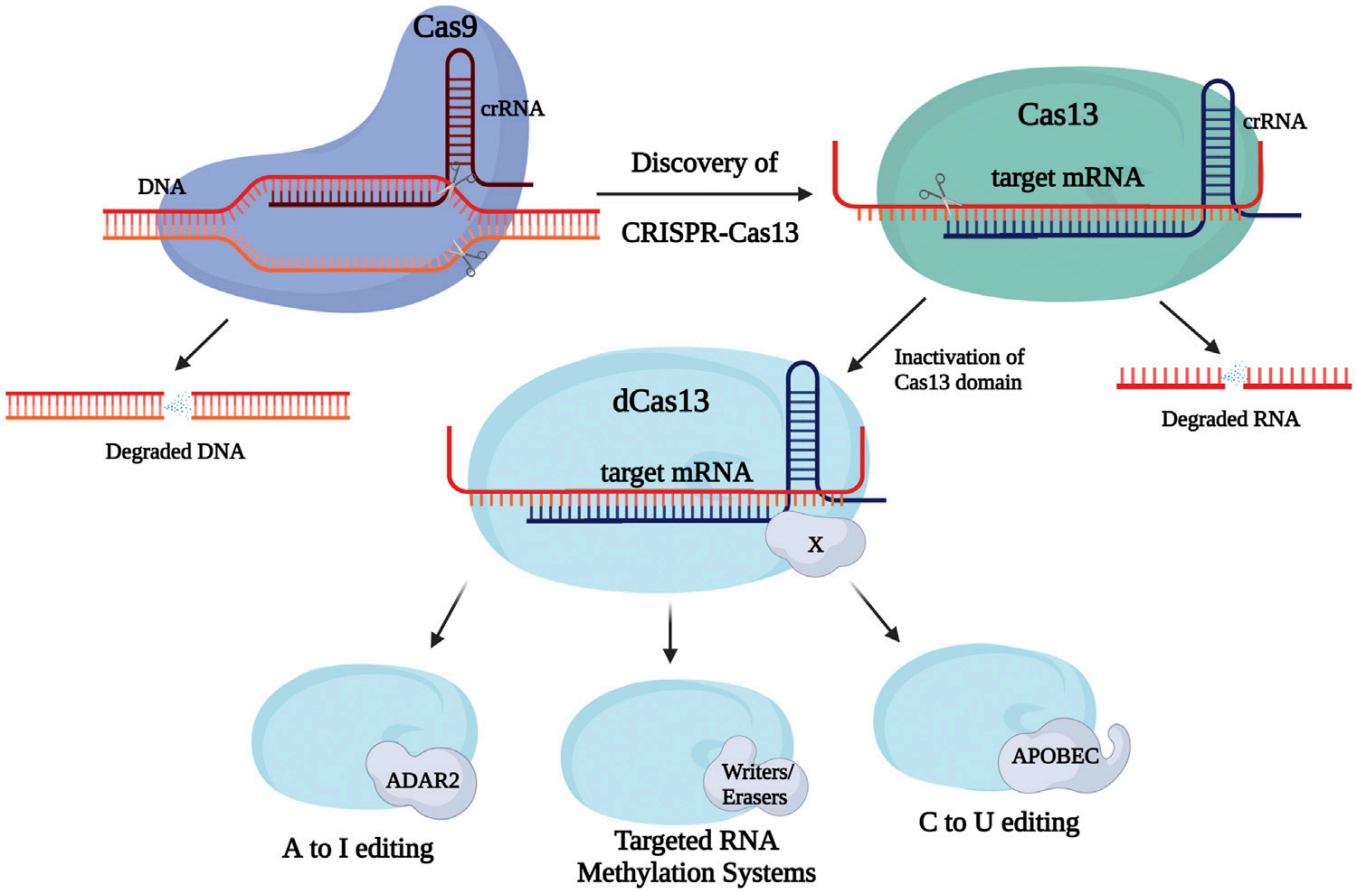
Discovery and development of Clustered Regularly Interspaced Short Palindromic Repeats (CRISPR)/Cas systems for epitranscriptome editing. CRISPR-Cas9 is established as a tool capable of DNA targeting and modification, however, the rising implementation of RNA editing strategies led to the discovery of a natural RNA-targeting CRISPR system, Cas13. In turn, further modification of members of the Cas13 family in addition to fusions with different enzyme domains (e.g., writers, erasers) allows for performance of a variety of functions/modifications upon binding to its targets.

However, CRISPR-Cas9 was still prone to relatively major off-target effects, even with the help of protospacers to increase specificity (Gupta and Musunuru, 2014). This became an increasing concern when considered for use in RNA-targeted manipulation. The retention of DNA-targeting activity itself would likely increase the chance of further unwanted off-target effects (Perculija et al., 2021). A possible solution to this dilemma would appear through the more recent investigations of RNA-targeting CRISPR-Cas13 systems (Figure 1).

Type VI (Cas13) systems are used in prokaryotes to target and ultimately cleave RNA. Systems such as Cas13a and Cas13b, which have different cleavage preferences, and guide CRISPR RNA (crRNA) structures (Perčulija et al., 2021), have been incorporated in several editing constructs (Table 1). For example, the RNA Editing for Programmable A to I Replacement, version 2 (REPAIRv2) tool—composed of inactive Cas13b (dCas13) and a mutant ADAR2 deaminase domain, edits adenosine to inosine, and making it potentially useful for treating diseases derived from G to A mutations (Cox et al., 2018). dCas13 with an APOBEC domain allows for cytidine to uridine edits, while a further modified version of REPAIRv2 called RESCUE edits C–U, while keeping the original ADAR2 deaminase activity intact (Cox et al., 2018; Abudayyeh et al., 2019). Interestingly, the REPAIR systems are able to encode an exact target site into its guide due to dCas13 having no targeting sequence restrictions, giving it the capacity to target any adenosine in the transcriptome. Even though efficiency is relatively high in these systems (up to 30% for REPAIRv2 and ∼70% percent for RESCUE), off-target events are still substantially present (Abudayyeh et al., 2019). Nevertheless, rational mutagenesis has been able to increase the specificity dCas13b-ADAR_DD_ complexes by more than 900-fold. A further subclass of Cas13b proteins known as Cas13bt has recently been constructed into variant REPAIR and RESCUE editors for transcript knockdown. Cas13bt′s smaller size permits packaging of the editor into an adeno-associated virus for delivery (Kannan et al., 2021).

**TABLE 1:** Comparison of various RNA-targeting systems for base editing.

Name	Components	Function	Advantages	Relative efficiency	Off-target	References
REPAIRv2	dCas13b + mutant ADAR2_DD_	A to I	AAV packaging for delivery	∼30%	High	Perčulija et al. (2021)
RESCUE	dCas13b + ADAR2_DD_	C to U	Original ADAR2_DD_ activity maintained; can target any adenosine in genome	∼70%	High	Cox et al. (2018); Abudayyeh et al. (2019)
Cas13bt	Cas13bt1 and Cas13bt3 + ADAR2_DD_	A • I;C • U	Smaller size allows for AAV delivery	∼40–50%	Medium	Kannan et al. (2021)
CasRx	dCas13X.1 + ADAR2_DD_	A • I;C • U	Allow easier *in vivo* delivery	High	Low	Konermann et al. (2018)
CIRTS	Effector protein + RNA hairpin binding protein + ssRNA binding protein	Flexible	Small size; minimal immune response	High	Low	Rauch et al. (2019)
REWIRE	RNA-recognizing PUF domain + variable deaminase domain	A to I C to U	Target extranuclear genes *In vivo* AAV delivery Operate independently of endogenous repair pathways	∼60–80%	Low	Han et al
λN–BoxB	ASO gRNA + λN proteins + ADAR2_DD_	A to I	Improved *in vitro* editing with A•C mismatch	Medium	High	Montiel-Gonzalez et al. (2013)
RESTORE	Endogenous ADAR1 + ASO gRNA	A to I	Requires only oligonucleotide administration (for ADAR recruitment)	∼75–85%	Low	Merkle et al. (2019)

Various Cas effector complexes have been shown to be proficient at targeting several other types of RNA modifications. CRISPR-Cas13d variants such as CasRx have been engineered for knocking down endogenous RNA as well as controlling RNA alternative splicing. Specifically, the inactivation of the HEPN-mediated RNase activity on dCasRx allows for flexible RNA-binding and specific targeting of RNA elements (Konermann et al., 2018). In addition, two compact families of Cas13 ribonucleases—Cas13X and Cas13Y—were identified from microbes. From these systems, Cas13X.1 was designed to perform RNA interference in mammalian cells with high levels of efficiency (Xu C et al., 2021). Furthermore, dCas13X.1 was combined with ADAR2_DD_ in order to generate various RNA base editors, namely A to I (xABEs) and C–U (xCBEs) base editors, which were capable of editing at various mammalian loci. Subsequently, truncations of dCas13X.1 generating mxABEs and mxCBEs overcame the size limitations other Cas13 systems faced for *in vivo* delivery. The xABE and mxABE editors were found to perform A–I conversions more efficiently than the REPAIR systems when paired with a crRNA guide, and the mxCBE systems were found to outperform the RESCUE systems for C–U editing, demonstrating high transcriptome fidelity and reducing off-target edits (Xu C et al., 2021)

### Emergence of Programmable CRISPR/Cas RNA Methylation Tools

**FIGURE 2:**
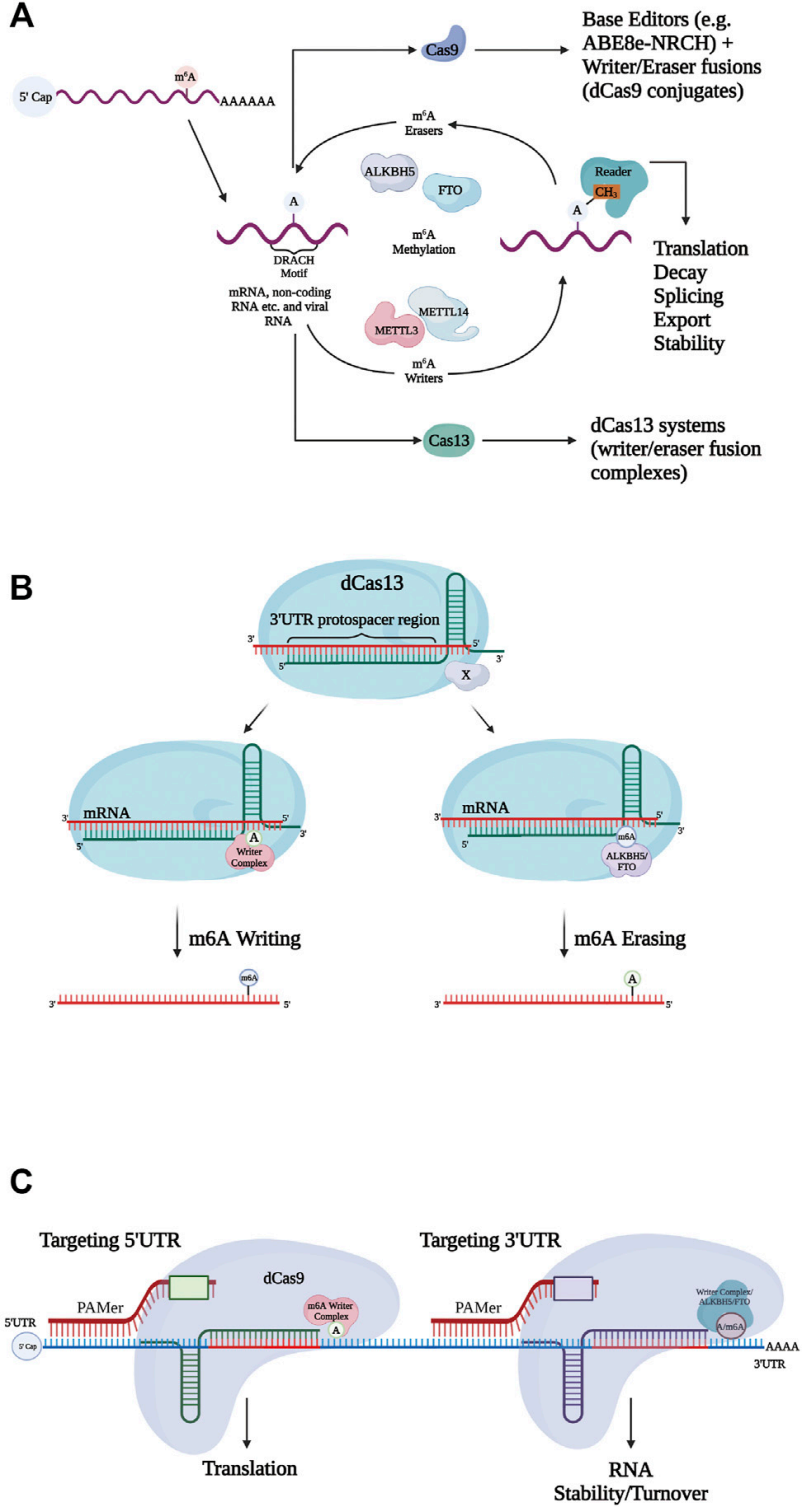
Mechanisms for N6-methyladenosine (m6A) regulation. **(A)** Writers and erasers tightly regulate the presence of m6A on transcripts, by targeting the m6A motif (DRACH). m6A is recognized by readers, initiating steps regulating mRNA stability, translation etc. Modification systems can be expanded to include both Cas9 (base editors, writer/eraser fusions) and Cas13 ((de)methylation systems). **(B)** Catalytically inactive Cas13 (dCas13) fused to writer and eraser domains install and remove m6A modifications respectively. Single guide RNAs (sgRNAs) target specific sites (e.g., 3′UTR protospacer) for mRNA binding. **(c)** Catalytically inactive Cas9 (dCas9) conjugates fused to writer and eraser domains. Specific sgRNAs allow individual 5′UTR and 3′UTR targeting. Resulting effects of installing/erasing at the different UTRs vary. PAMer provides the NGG PAM sequence.

mRNA is subject to several post-transcriptional modifications (e.g., capping, adenylation) before undergoing translation. To date, m6A modifications are observed to be the most abundant type of endogenous mRNA modification in eukaryotes (Wilson et al., 2020). Many m6A modifications often play a vital part in physiological processes and are involved in the progression of malignancies such as human cancers (Jiang et al., 2021). They undergo dynamic regulation and can be removed (erased) and installed (written) by RNA methylation complexes in order to observe the effects on specific pathways and systems (Figure 2A). Targeted RNA methylation (TRM) systems—composed of catalytically inactive Cas13 (dCas13) fused with a methyltransferase domain—are capable of highly specific m6A installation on transcripts, and through such changes, mediate processes such as alternative splicing, transcript abundance, and translational efficiency (Figure 2B). Recent studies have explored two main TRM m6a writing systems, dCas13-M3 (methyltransferase-like 3) and dCas13-M3M14 (methyltransferase-like 3 and methyltransferase-like 14). Each was designed to be localized to the nucleus via a nuclear localization signal (NLS) or exported for cytoplasmic function via a nuclear export signal (NES). While both dCas13-M3nls and dCas13-M3M14nes were shown to have high, comparable on-target m6A installation efficiency, dCas13-M3nls was observed to be less prone to off-target edits than dCas13-M3M14nes and another explored editor, M3M14-dCas9. This particular difference was attributed to a truncated methyltransferase domain within dCas13-m3nls resulting in a lack of a METTL14 RNA-binding domain (Wilson et al., 2020). Interestingly, the nuclear localization of dCas13-M3nls has also been shown to cause no loss of translation efficiency after targeting 5′UTRs, and transcripts can be further regulated by other writers after modification (Wilson et al., 2020). Therefore, dCas13-M3nls was deemed overall the most practical TRM system within the nucleus for inducing m6A-mediated phenotypes.

dCas13 effectors are not the only CRISPR systems capable of m6A modification. CRISPR-Cas9 conjugates were coupled with single-chain methyltransferase and ALKBH5/FTO in order to form writers and erasers, respectively. The target site specificity of these Cas9 editors was programmable through guide RNA (Figure 2C). Specifically, installation at the 5′UTR allowed for non-canonical translation, while erasure and installation at the 3′UTR influenced RNA turnover (Liu et al., 2019). It is still relatively unclear as to how the individual localization of the writer and eraser proteins (nucleus or cytosol) may affect TRM editing in different systems.

The reversibility of m6A modifications can be dynamically regulated by m6A demethylases such as ALKBH5 and FTO. It, too, is capable of targeting mRNA (for demethylation) when combined with dCas13 and targeted with an sgRNA in order to form a dm6ACRISPR system (Li et al., 2020). This construct has been used for *in vivo* manipulation of oncogenic targets on EGFR and MYC transcripts for controlling cell proliferation, while limiting the number of off-target edits. Demethylation efficiency was increased by calibrating the distance between the target sites and methylated sites to 100–300 nucleotides (Li et al., 2020). However, the effects of dm6ACRISPR demethylation vary due to the activity of different reader proteins, as it was observed that methylation of CYB5A and CTNNB1 transcripts resulted in increased mRNA stability, and thus increased expression. While this was one of the first systems set out to establish the role of m6A demethylation with respect to overall cell function (Li et al., 2020), more recent studies have explored a similar system with NLS CasRx, which is the smallest and most efficient of the Cas13 family for RNA knockdown. Thus, it was speculated that binding erasers (ALKBH5) and writers (e.g., METTL3) to dCasRx would allow for specific site manipulation on par with dCas13b (Li et al., 2020; Xia et al., 2021). In addition, its smaller size would allow for easier delivery into cells as a lentivirus, allowing for pooled screening approaches and widespread use in difficulty to transfect cell lines and primary cells (Li et al., 2020; Xia et al., 2021). We anticipate these programmable m6A tools will provide a functional platform to interrogate site-specific m6A RNA modifications that contribute to a wide range of physiological processes and complement existing m6A profiling studies.

### Transcriptome and Epitranscriptome CRISPR Screening Approaches

The development of high-throughput technologies and genome-editing has revolutionized the field of functional genomics, which attempts to assess the function and interaction of genes in a systematic approach. Screening tools such as short interfering RNAs (siRNAs) were effective and consistent at silencing gene expression for genetic screens, however, both the cost, the short life of the siRNAs, and the lack of efficient delivery into primary cell cultures put limits on its application (Bernards et al., 2006). On the other hand, short-hairpin RNAs (shRNAs) were able to maintain constant levels of silencing after vector delivery, and their compatibility with different types of vectors allowed for delivery into a greater variety of cells (Bernards et al., 2006). More recently, CRISPR-Cas13 has been explored as an alternative screening tool of shRNAs. Since Cas13 based TRM systems have only recently been characterized, screening has not been extensively applied towards evaluating m6A modifications. We anticipate TRM screening platforms to emerge in the near future, which will incorporate principles of Cas13-based screens that have investigated linear genes, and more recently non-coding RNAs such as circular RNAs (circRNAs).

Through the use of CRISPR-Cas13d in combination with improved designs of sgRNAs for circRNA back-splicing junction (BSJ) sites, circRNA silencing specificity is increased, indicating its effectiveness for high-throughput screening of functional circRNAs (Zhang et al., 2021). In a side-by-side comparison of Cas13d and shRNA functional screens, the read distribution for both gRNAs and shRNAs were found to both be highly correlated. However, non-targeting controls of shRNAs yielded more false-positive results compared to the gRNAs of Cas13d, indicating Cas13 to be a more refined method for circRNA targeting (Zhang et al., 2021). This difference was attributed to the off-target effects of shRNAs, while at the same time establishing on-target specificity of Cas13d. Like TRM systems, Cas13d (CasRx) is capable of being optimized for compartmental distribution. CasRx-NLS was observed to be optimal for circRNA targeting in the nucleus, while lack of the NLS signal optimized CasRx targeting of cytosolic circRNAs, allowing a further advantage over shRNAs (Zhang et al., 2021). This ability to compartmentalize allows CasRx systems to outperform RNAi. While efficiency between CasRx and RNAi is comparable, RNAi is not capable of compartmentalizing (Wessels et al., 2020), and is subject to more off-target effects (Zhang et al., 2021). CasRx is currently one of three main effector proteins—along with *Pgu*Cas13b and *Psp*Cas13b—that have been identified, however, CasRx was shown to be consistently more effective at target RNA knockdown, even more so when fused with an NLS (Wessels et al., 2020). CasRx-BSJ-gRNA systems are applicable for genome-wide screening, in particular for observing the loss-of-function effects of circRNAs originating from the gene’s internal exons (Zhang et al., 2021)*.* We envision similar approaches will utilize CRISPR/Cas programmable RNA modification tools (as discussed earlier and below) to study the epitranscriptome through pooled screening, which will serve as powerful tools to assess all types of RNA modifications.

### Base Editors

Base editors usually indirectly modify RNA transcripts by modifying the DNA, thus, off-target edits are issues for which there are no possible solutions. It was hypothesized that embedding editing enzymes such as APOBEC1 and Tad-TadA into the middle of nCas9 instead of linking it to its N-terminus would reduce the off-target effects (Liu et al., 2020). Cas-embedding would introduce steric effects that could possibly block off-target editing. In combination with other techniques such as the usage of short-rigid linkers, the editing window can be narrowed for increased specificity (Liu et al., 2020). RNA base editors have been able to benefit from this technique as well. Off-target effects were found to be slightly reduced when the ADAR2_DD_ was embedded into dCasRx’s flexible loop instead of being linked at its terminal (Liu et al., 2020). Altering the structural components of the base editors has been shown to increase efficiency as well. Manipulating Cas9’s secondary structure improved the interactions between the Cas9 endonuclease and the other base editor components in order to lower the level of off-target RNA editing. The same ABE variant was shown to behave differently with RNA and DNA through individual secondary structure changes (Nguyen Tran et al., 2020).

Some enhancements to base editor systems that seem to broaden their function could perhaps introduce a novel approach to future base editing techniques. Usually, CRISPR base editors are capable of modulating only one type of base modification (e.g., ABEs, CBEs). Grünewald and colleagues (Grünewald et al., 2020) were able to design a dual function base editor derived from miniABEmax-V82G and Target-AID deaminases called synchronous programmable adenine and cytosine editors (SPACE), capable of synchronous A-to-G and C-to-T edits (Grünewald et al., 2020). The editing window of SPACE is narrower compared to miniABEmax-V82G and Target-AID, however, it does not seem to provide an additional editing efficiency advantage. SPACE does have comparable (if not lower) efficiency capabilities to the individual base editors themselves, while minimizing off-target effects (Grünewald et al., 2020). This seems to be consistent with another set of designed dual-function editors. Target-ACE, Target-ACEmax and ABCEmax are composed of cytidine, and adenosine deaminases bound to nCas9 (Sakata et al., 2020). Like SPACE, Target-ACEmax was found to possess on and off-targeting capabilities like those of the single-function base editors. However, Target-ACEmax and ABCEmax were found to be useful as genome editing tools for applications such as therapeutics, which are capable of higher delivery efficiency. In particular, Target-ACEmax was able to mediate heterologous base editing more efficiently than current systems such as CRISPR-X (Sakata et al., 2020).

While *in vitro* studies were undertaken for optimizing base editor efficiency, there have been steps taken for using base editing *in vivo*, specifically in non-human primates and mice (Rothgangl et al., 2021). Through lipid nanoparticle-mediated (LNP) delivery, an ABE-encoding nucleoside-modified mRNA combined with modified gRNA was capable of editing PCSK9 in macaque livers (30 percent efficiency) with few off-target edits, resulting in lower LDL cholesterol. It was hypothesized that the efficiency rates mediated by LNP delivery would allow for treatment of other genetic liver diseases (Rothgangl et al., 2021). As an extended ABE presence was thought to eventually result in an increased number of off-target edits as well as induce an immune response, the treatments were delivered in doses. With each repeated dosage, the editing rates were found to increase in mice, but not the macaques (Rothgangl et al., 2021). Therefore, future adjustment of the dosage could possibly lead to increased editing rates in macaques, allowing this approach to eventually be applied in humans (Rothgangl et al., 2021).

Non-CRISPR based tools have shown promise for base editing in human cells with lower off-target effects. REWIRE (RNA editing with individual RNA-binding enzyme) is a gRNA independent system derived from human proteins (Han et al). It is a one-enzyme technique, which eliminates any complications that may arise from assembly. It can edit without involving endogenous repair pathways, which extends the possibility of personalized therapy to post-mitotic cells such as neurons. REWIRE is not only limited to nuclear compartments, as it was also found to be capable of targeting mitochondrial genes. Despite its capabilities, it is still subject to significant off-target effects due to the enzyme’s PUF domain’s naturally small target. However, this can be mitigated by increasing the number of PUF repeats or by modification to its other domains (e.g., deaminase). In theory, PUF can therefore also be applied for other purposes including RNA methylation with high specificity and increased targeting capabilities if associated with domains such as the methyltransferase domain of METTL3.

The principles of CRISPR-Cas systems have also been applied to the design of CIRTS (CRISPR/Cas inspired RNA targeting system). These endogenous transcriptome editing tools separate the main required functions (e.g., selective hairpin binding, gRNA complementary to the target and effector protein) that Cas13 holds in one protein domain into a complex of several different proteins, each one responsible for a single function (Rauch et al., 2019). There are advantages to separating the functions amongst several domains. First, the overall complex itself is smaller than the current CRISPR/Cas modification systems, allowing for easier direct protein delivery. As well, the individual proteins themselves do not have to be CRISPR/Cas derived, as human proteins can be substituted to engineer a CRISPR-Cas13 system. Importantly, from a therapeutic stance, it may be possible to edit RNA without inducing an immune response (Rauch et al., 2019).

Another practical method is the use of antisense oligonucleotide (ASO), which can modulate RNA expression, and have been under development as therapeutic tools for years. In systems such as recruiting endogenous ADAR to specific transcripts for oligonucleotide-mediated RNA editing (RESTORE), only delivery of the oligonucleotide is required. The RESTORE system has been found to achieve higher efficiency than its Cas13 counterparts with limited off-targeting (Merkle et al., 2019). On the other hand, systems such as the λN–BoxB RNA interaction requires binding between a λN protein and a BoxB hairpin loop containing the ASO (Montiel-Gonzalez et al., 2013). Base editing activity comes from the endogenous ADAR2 domain that binds to the λN protein. Potentially, ASO may be able to modulate RNA in the context of m6A methylation by blocking known sites where m6A readers and writers bind to.

### Dissecting m6A Modifications With Base Editors

Due to the results and successes of base editor approaches, more recent studies have begun to utilize base editing technology to study m6A modifications. In order to observe the effects of m6A modifications on miRNA and long non-coding RNA, an adenine base editor system (ABE7.10) was used to induce single site base change. After the targeted mutation of an m6A site (T-A conversion) upstream of miR-675 in the H19 locus in HEK293T cells, miR-675, and H19 expression were observed to be suppressed, resulting in an increase in apoptosis (Hao et al., 2020). It was hypothesized that the reduced expression resulted in an increased presence of p53 protein, thus inducing cell death which indicates the role of m6A in regulating miR675 and H19 expression (Hao et al., 2020), and by extension, cell survival. As stated previously, m6A is highly involved in cancer development. This was further supported by (Lee et al., 2021), who showed the upregulation of METTL3 activity and m6A frequency commonly found in cancers. In particular, the methylation of homeobox containing 1 target mRNA has been linked to loss of p53 signaling and issues with telomere regulation (Lee et al., 2021).

Aside from direct RNA modifications and genome editing, base editors have been leveraged for genome-wide screening as well. Unlike canonical Cas9 knockout screens, iBARed cytosine base editing-mediated gene knockout (BARBEKO) systems do not utilize double-strand breaks for gene knockouts (Xu P et al., 2021). Instead, knockout methods are directed to the start codons and splice sites of target RNA, as well as stop codon introduction. Associated sgRNAs are designed to carry internal barcodes (iBARs), which assist with screening efficiency (Xu P et al., 2021). Another advantage found over Cas9-mediated cleaving is attributed to the absence of double-strand break activity. Inducing double-strand breaks in amplified regions often leads to false positives, however, BARBEKO is not subject to such copy-number effects. Along with being more cost and labour efficient, BARBEKO was found to be ideal for high-quality screening primary cells as well as *in vivo*, despite the usual risk of DNA damage and small sample sizes (Xu P et al., 2021). Currently, a method is being explored to mediate potential effects of BARBEKO screens with high multiplicity of infection, as the lentiviral transduction of several sgRNAs may cause cytotoxicity. It is quite likely that a similar system to BARBEKO could be used to study m6A through disrupting the canonical m6A RNA motif.

## Therapeutic Outlook for CRISPR/Cas Systems and m6A

### Therapeutics

m6A has been shown to be associated with many physiological and pathological processes. However, it is only one of many different types of modifications which can be targeted. As such, the possible use of CRISPR and base editors as tools for therapeutic processes has been explored and is currently under promising development.

**FIGURE 3:**
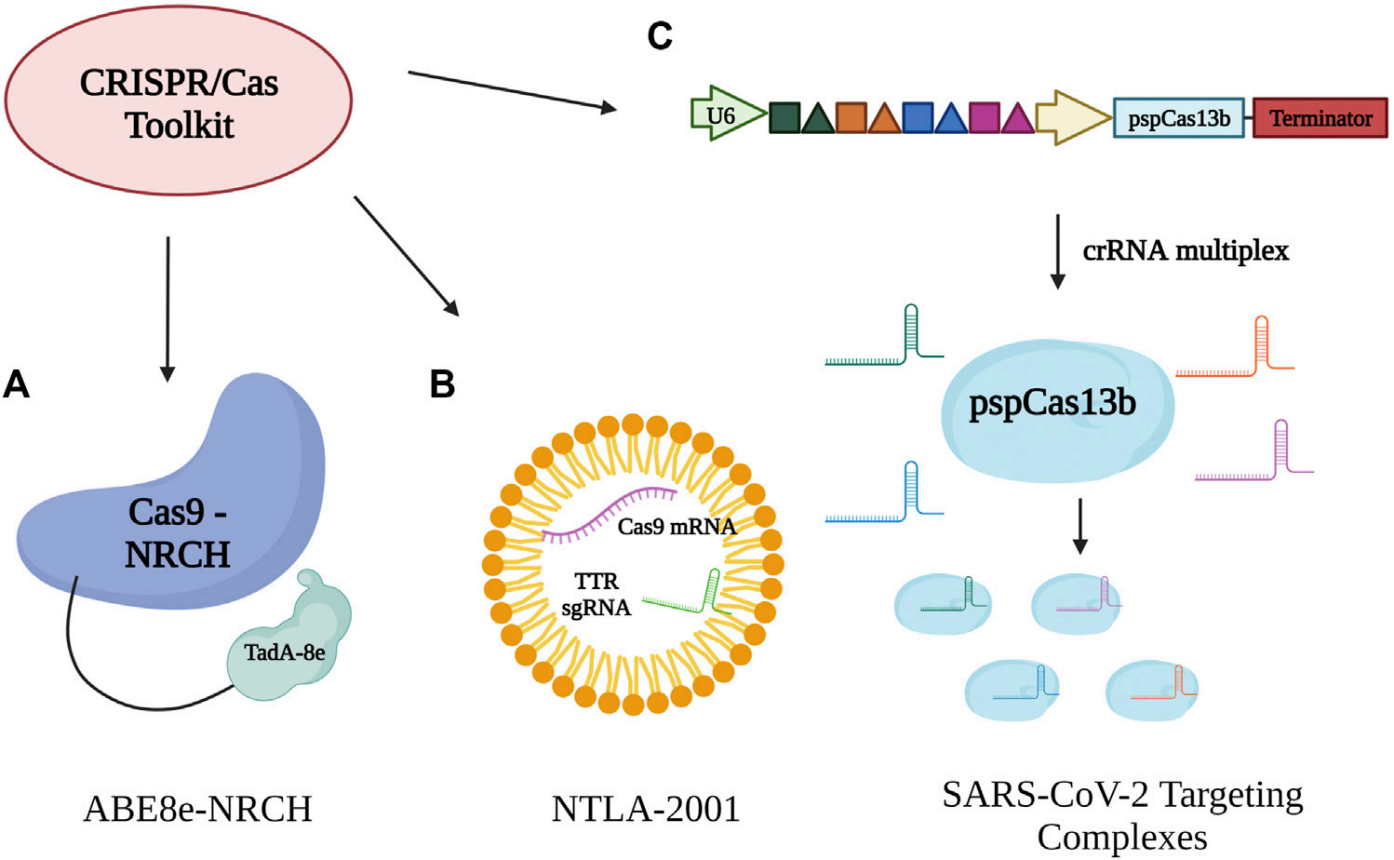
Therapeutic applications for CRISPR/Cas systems. **(A)** A specialized adenine base editor composed of Cas9-NRCH bound to a TadA-8e domain. ABE8e-NRCH converts the pathogenic variant of the sickle cell disease allele into a non-pathogenic variant. **(B)** Lipid nanoparticle delivery of Cas9 mRNA and transthyretin-targeting (TTR) gRNA for treatment of ATTR amyloidosis with the goal of reducing both mutant and wildtype levels of transthyretin protein. **(C)** SARS-CoV-2 targeting complexes. A set of four targeting crRNAs is utilized in combination with pspCas13b in order to reduce the virus’s chance for escape through mutation and daughter strain proliferation. Main targeted sites are conserved regions and sequences of coronaviruses.

One of the fields of research is sickle cell disease. A customized base editor—ABE8e-NRCH - was developed in order to convert the sickle cell disease allele into a non-pathogenic form (Makassar beta-globin) (Figure 3A). Transplantation of human hematopoietic stem cells indicated long-lasting gene editing of up to 80% in mice, although it was shown that only about 20% was required for phenotypic rescue after autologous treatment (Newby et al., 2021). Using the base editor was ultimately more efficient than other techniques such as induction or lentiviral expression, which would have left the sickle cell allele untouched. It also avoids the possible faults of direct Cas9 application, such as larger genome deletions as well as possibly cell death through inappropriate p53 activation (Newby et al., 2021). An additional advantage of using base editing is the lack of requirement for DNA delivery, a usual component of other gene therapies which could lead to insertion mutations and other toxic effects. As the treatment is only required once, it also lowers the effects of double-strand breaks (Newby et al., 2021). The main concern regarding base editors is the possibility of off-target edits. However, these were kept to a minimum through a “CACC” PAM, and the changes to off-target sites were observed to be of little to no consequence. Possible methods for improving the safety and effectiveness of this base editor therapy involve pairing different Cas9 variants and deaminase domains in order to minimize off-target edits, as well as dose, and delivery optimization (Newby et al., 2021).

Though base editors seem to be ideal tools for therapy, CRISPR-Cas9 itself for *in vivo* gene editing is by no means an inferior method. LNP delivery of Cas9 endonuclease mRNA and transthyretin-targeting gRNA (NTLA-2001) (Figure 3B) was proposed as a treatment for ATTR amyloidosis. Current treatments require constant and long-term administration for RNA knockdown; however, the disease still progresses (Gillmore et al., 2021). The liposome−polycation−pDNA (LPD) method works through a dose-dependent effect, which is currently in the process of being escalated in order to reduce overall transthyretin (TTR) levels for both wild-type and mutant forms, with the hope of producing permanent knockdown after a single administration of treatment. As the liver cells are the main source of TTR, primary human hepatocytes were used for testing in order to increase efficiency and lower toxic effects (Gillmore et al., 2021). Cas9 off-target edits were not observed with NTLA-2001 and any induced genome variation through editing was deemed of little risk, thus setting precedent for predictable outcomes *in vivo* (Gillmore et al., 2021)*.* Additional preclinical platforms are also being explored, such as prime editors which fuse a reverse transcriptase domain to dCas9, and thus facilite genome knock-in to rescue protein expression in mammalian cell lines (Anzalone et al., 2020).

### METTL3-Mediation for Viral Detection

The detection of pathogens such as RNA viruses—in particular Vesicular Stomatitis Virus—is shown to have closely involved METTL3 methyltransferase. METTL3 was observed to translocate to the cytoplasm and increase m6A modification levels on viral transcripts (Qiu et al., 2021). This led to reshaping of the RNA, causing the reduction in double-stranded RNA formation—a key antiviral signal—thus lowering sensitivity and innate immune signaling and response. However, the identification of METTL3 as an innate suppressor has made it a possible target for reinstating immune response against viral infections and even curing patients. This would be a promising method especially for tumour suppression and immunogenicity, which is heavily dependent on innate immunity activation (Qiu et al., 2021).

### Stopping SARS-CoV-2 Replication

In alignment with the current events of SARS-CoV-2, CRISPR-Cas13b has been modified in an attempt to prevent the virus’s replication. One of the dangers of the virus is the development of strains with variation in transmissibility and pathogenic effects. CRISPR-pspCas13b was utilized along with two methods in order to account for the possibility of viral mutation. Multiple crRNAs were utilized (Figure 3C) in order to maximize accessibility to the viral RNA, as well as limit the virus’s options for escape through mutation (Fareh et al., 2021), similar to that of a drug cocktail. Unlike other various viral inhibitors, generating the appropriate crRNAs for pspCas13b is a specific, and efficient process. As well, pspCas13b possesses a specific characteristic—a positively charged central channel—that allows it to function even with some mismatched nucleotide pairing. Ultimately, this increases the use of the associated crRNA to suppress both the parental virus and future variants (Fareh et al., 2021). Other technical strategies have been employed concurrently, such as targeting conserved regions for coronaviruses in order to further reduce the chance of mutational escape, even with only a single crRNA present. Targeting was also considerably calibrated in order to limit the possibility of off-target effects on human transcriptomes (Fareh et al., 2021). Due to its flexibility in design, it is expected that CRISPR-Cas13 will be an overall efficient tool against viral pathogenesis because it is more difficult for strains to evade compared to more traditional antiviral therapeutics.

### Perspectives

CRISPR/Cas systems and base editors are shown to be overall useful and proficient modification tools. However, one of the persistent issues being faced is the level of off-target editing. Although the rate is observed to be lower than that of previous genome editing systems, improvements can be made; one of the major strategies involves combining variants of CRISPR/Cas systems with other editing domains and gRNAs to optimize targeting and binding efficiency for each system’s purpose. Attaining accurate targeting should be one of the more important steps for further *in vivo* application of base editors and CRISPR/Cas systems in therapeutics. As m6A is an abundantly occurring modification as well as a common player in many pathological processes, one would expect that several editing therapies in the future will likely revolve around these sites.

In particular, RNA targeted methylation systems should be useful for treatment of viruses and pathogens. As methylation often plays a necessary role for the proliferation of RNA viruses, it thus provides a feasible target for treatment. For example, SARS-CoV-2 mRNA is methylated by an nsp16/nsp10 enzyme complex at the 2′-OH site of its first nucleotide in order to alter the composition of its cap, thus rendering it immune from surveillance (Viswanathan et al., 2021). Targeting this specific methylated site (perhaps with an eraser TRM system) would make a promising first step in a potential series of processes for treating SARS-CoV-2.

Although (CRISPR) RNA-targeting is the more current and popular methodology for phenotypic observation and novel therapeutic approaches, genome-targeting itself is still a very viable option. Recently, delivery of Cas9 mRNA with herpes simplex virus type 1 (HSV-1) erasing lentiviral particles (viral-targeting gRNA) stopped HSV-1 viral proliferation, as well as eradicated any latent viral reserves (Yin et al., 2021). An added advantage to this approach was the noted absence of off-target effects.

For industrial purposes, DNA and RNA targeting systems will likely play a prominent role in agricultural production. Like humans, m6A modifications are critical in plants. In early trials, it was shown that demethylation of m6A by FTO demethylase resulted in increased levels of yield and biomass for rice and potato crops (Yu et al., 2021). What m6A demethylation accomplished was elevating the amount of poly (A) RNA as well as the degree of open chromatin (thus influencing the levels of gene transcription). It is likely that m6A demethylation will be applicable for yield improvement in other agricultural plants in the future, but also will lead to exploring how m6A is involved in transcription in plants.

It is known that m6A modifications can affect RNA stability, and therefore increase or decrease the amount of translation that occurs as well as the longevity of the molecule itself. Therefore, introducing m6A modifications on individual RNA species could serve as an approach to fine-tune the stability of RNAs, which could have applications for RNA vaccine and RNAi therapeutics. In addition, engineering TRM systems for *in vitro* m6A detection of pathogenic transcripts will help preclinical research studies and serve as a blueprint for further extended research into other RNA modifications. We expect CRISPR-based approaches will allow the study of the modifications endogenously, as opposed to using conventional exogenous approaches that rely on luciferase reporter assays, which are currently used to measure the role of RNA modifications on protein stability. It is foreseeable that further exploration will bring us to the stage where we are eventually understanding the functions of individual m6A sites, by introducing a variety of writer and eraser fusions that assist with various biological studies, and optimizing CRISPR complexes for high quality pooled screens.

Costwise, current base editors, CRISPR, and techniques such as CIRTS and RESTORE are more economical than their predecessors. While base editors in general can be delivered in a variety of ways and provide an opportunity for the pursuit of personalized medicine, toxicity is still a concerning obstacle especially for systems such as BARBEKO that rely on sgRNA delivery. Overall, the major transition to Cas systems—especially CRISPR/Cas13—in search for a solution for programmable RNA editing was a fruitful process. Editing efficiency was improved and off-target effects were generally reduced, although they currently remain higher than desired. As more CRISPR proteins are being discovered, we expect that ones with smaller size and lower off-target efficiency will be available to address these shortcomings. With the right coupling, both Cas9 and Cas13 conjugates can target methylation sites for writing/erasing, and the same applies to PUF systems as well. PUF complexes are capable of targeting multiple subcellular compartments, although off-target effects need to be improved by modifying the PUF domain. On the other hand, off-target effects are less of a pressing issue for ASO systems. By adjusting the ADAR domain of ASO systems, the editing efficiency can increase. However, the risk of off-target editing also increases, which is an area that requires further optimization.
